# Explainable machine learning for predicting ICU mortality in myocardial infarction patients using pseudo-dynamic data

**DOI:** 10.1038/s41598-025-13299-3

**Published:** 2025-07-31

**Authors:** Munib Mesinovic, Peter Watkinson, Tingting Zhu

**Affiliations:** 1 Department of Engineering Science, University of Oxford, Oxford, UK; 2 Nuffield Department of Clinical Neurosciences, University of Oxford, Oxford, UK

**Keywords:** Explainability, Icare, Machine learning, Myocardial infarction, Prediction, Computational biology and bioinformatics, Health care, Engineering

## Abstract

Myocardial infarction (MI) remains one of the greatest contributors to mortality, and patients admitted to the intensive care unit (ICU) with myocardial infarction are at higher risk of death. In this study, we use two retrospective cohorts extracted from two US-based ICU databases, eICU and MIMIC-IV, to develop an explainable pseudo-dynamic machine learning framework for mortality prediction in the ICU. The method provides accurate prediction for ICU patients up to 24 hours before the event and provides time-resolved interpretability. We compare standard supervised machine learning algorithms with novel tabular deep learning approaches and find that an integrated XGBoost model in our EHR time-series extraction framework (XMI-ICU) performs best. The framework was evaluated on a held-out test set from eICU and externally validated on the MIMIC-IV cohort using the most important features identified by time-resolved Shapley values. XMI-ICU achieved AUROCs of 92.0 (balanced accuracy of 82.3) for a 6-hour prediction of mortality. We demonstrate that XMI-ICU maintains reliable predictive performance across different prediction horizons (6, 12, 18, and 24 hours) during ICU stay while also achieving successful external validation in a separate patient cohort from MIMIC-IV without any previous training on that dataset. We also evaluated the framework for clinical risk analysis by comparing it to the standard APACHE IV system in active use. We show that our framework successfully leverages time-series physiological measurements from ICU health records by translating them into stacked static prediction problems for mortality in heart attack patients and can offer clinical insight from time-resolved interpretability through the use of Shapley values.

## Introduction

Acute myocardial infarction (AMI) encompasses a spectrum of clinical presentations including ST-segment elevation myocardial infarction (STEMI), non-ST-segment elevation myocardial infarction (NSTEMI), and acute coronary syndrome with confirmed myocardial damage. myocardial infarction remains one of the greatest contributors to cardiovascular deaths in the world whose incidence remains critically high with approximately every 40 seconds someone in the United States suffering an episode^1^. Cardiovascular diseases (CVDs) also represent a major cost burden globally with MI in the ICU being one of the most common CVD-related conditions in the critical care system^2^. In 2015, there were more than 18 million CVD-related deaths with MI accounting for over 15% of overall mortality and research showing that healthcare costs skyrocket with longer and more inefficient treatment in the ICU^3–5^. Patients who exhibit MI are usually referred to the ICU, however, they are 10% more likely to suffer another episode in the days following and are at higher risk of death, especially the elderly^6^. For STEMI, NSTEMI, and general AMI patients admitted to the ICU, studies have found mortality rates as high as 17.6% but usually closer to 11% within the ICU in multicentre ICU studies^7–10^. Mortality prediction models can help with prioritising patients with myocardial infarction and supplant existing mortality prediction tools like the APACHE system deployed in US critical care centres, which has been criticised as too general and inaccurate for specific populations and diseases^11,12^. Others like the Framingham Risk and the GRACE score are simple linear risk calculators for general mortality for heart disease patients and do not support individual prognosis from longitudinal patient data^13,14^. Machine learning would allow us to provide individual prognosis while learning from complex longitudinal patient dynamics^15^. When machine learning is combined with interpretability methods, it can be a useful tool for clinical guidance and decision-making.

Recent machine learning approaches for MI mortality prediction have shown promising but mixed results. Lin et al. demonstrated that XGBoost and random decision forest algorithms achieved superior performance (AUROC 0.835) compared to traditional scoring systems like SOFA using MIMIC-IV data^16^, while nomogram-based studies report AUROCs of 0.846-0.885^17,18^. However, the aforementioned methods are fundamentally static and cannot capture dynamic physiological changes during ICU stay. For time-series analysis, deep learning approaches including LSTM networks and GRUs have been explored for ICU mortality prediction^19,20^, but face challenges including computational complexity, data requirements, overfitting with sparse measurements, and lack of interpretability essential for clinical decision-making. Recent tabular deep learning methods like TabNet and NODE attempt to bridge performance-interpretability gaps^21,22^, but their superiority over traditional methods on healthcare tabular data remains contested^23^. Tree-based models continue to outperform deep learning on tabular data with characteristics common in healthcare: moderate dataset sizes, mixed data types, and uninformative features, due to their robustness to noise and built-in feature selection mechanisms.

Due to the recent nature of proposed tabular deep learning models, research applying them to healthcare challenges has been limited, with only one recent paper examining TabNet for ICU mortality prediction^24^. Prior deep learning work has largely focused on general ICU populations rather than MI-specific patients^25,26^. Existing risk algorithms for MI patients like TIMI and GRACE are simple linear models that ignore longitudinal physiological trajectories and provide no dynamic risk estimates during ICU stay^27,28^.

To address these limitations, we propose XMI-ICU (XGBoost for Myocardial Infarction in the ICU), a novel pseudo-dynamic machine learning framework that transforms time-series ICU prediction into connected static prediction problems. Our key innovation lies in the sliding time window approach: instead of using complex sequential models like LSTMs, we extract summary statistics (mean and standard deviation) from physiological measurements within defined prediction horizons (e.g., all measurements from ICU admission until 6 h before the event of interest). Each physiological variable (such as systolic blood pressure, lactate, or heart rate) becomes two features per time window, its mean value and variability measure, creating a comprehensive but interpretable feature set. This approach enables gradient-boosted models like XGBoost to capture some temporal dynamics through these engineered features while maintaining the interpretability and robustness that tree-based models provide. The framework automatically adapts to different prediction horizons (6, 12, 18, or 24 h in advance), providing clinicians with flexible, dynamic risk assessment throughout the ICU stay while avoiding the computational complexity and ”black box” nature of deep learning alternatives.

Our contributions are as follows:

1. We propose XMI-ICU, a novel pseudo-dynamic framework that transforms time-series ICU prediction into connected static problems by extracting temporal summary statistics from physiological measurements within sliding time windows. This approach enables interpretable gradient-boosted models to capture temporal dynamics while achieving superior performance compared to both traditional risk scores and recent deep learning approaches.
2. We demonstrate robust external validation across two major ICU databases (eICU and MIMIC-IV) with consistent performance across multiple prediction horizons (6, 12, 18, and 24 h in advance), proving the framework’s generalisability across different healthcare systems and temporal scenarios.
3. We introduce time-resolved Shapley value analysis that tracks how feature importance evolves across different prediction horizons, revealing dynamic clinical insights about which physiological measurements warrant attention at different stages of critical illness. We verify XMI-ICU’s clinical significance through decision curve analysis and comparison with APACHE-IV, demonstrating practical benefits for ICU decision-making beyond traditional performance metrics.

## Results

### eICU

**Fig. 1:**
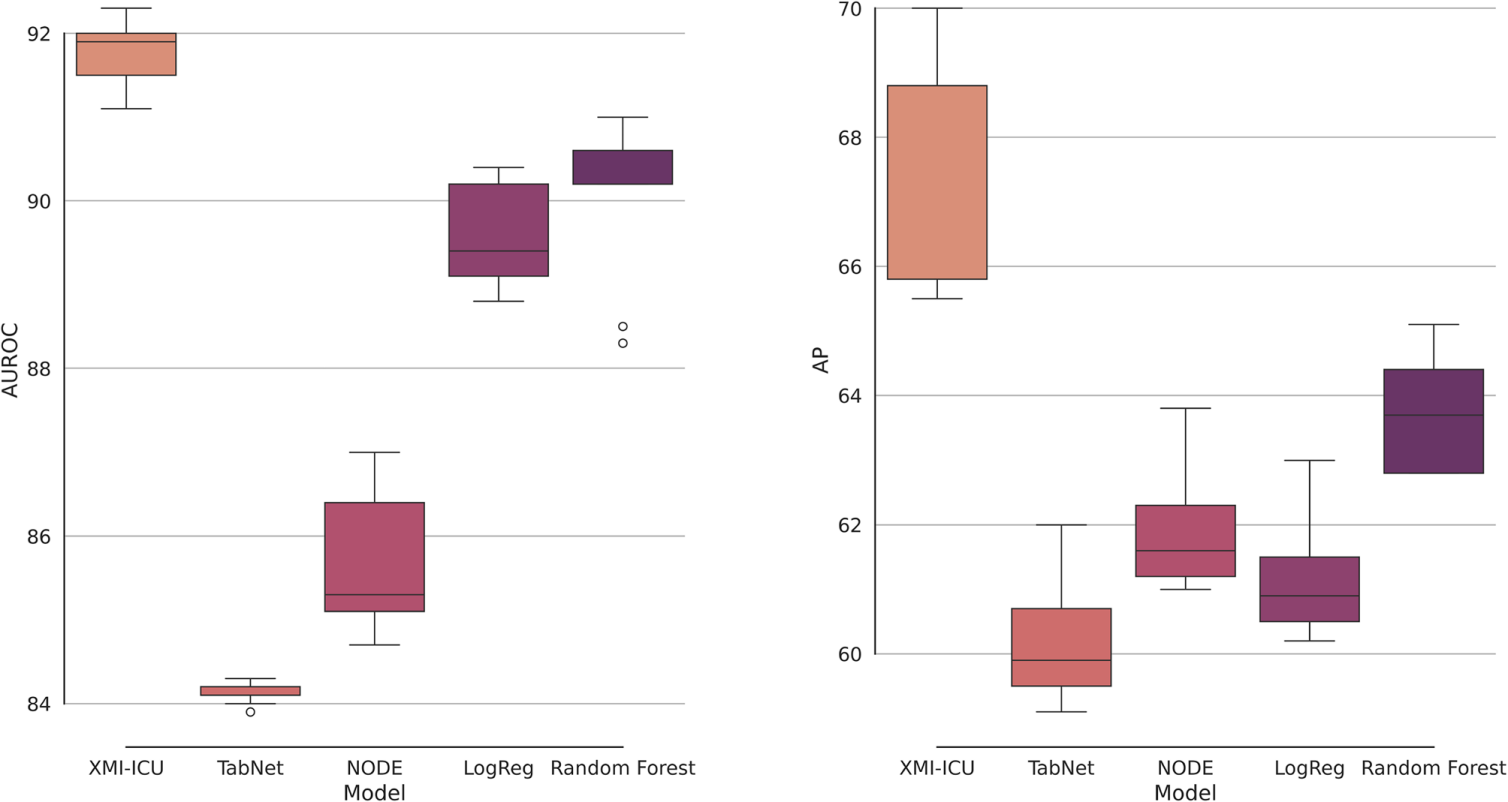
Evaluation performance of XMI-ICU to predict mortality 6 h in advance compared to other models for different metrics on eICU held-out test. AUROC and average precision results are a result of bootstrapping. The left figure represents the AUROC and the right the average precision results.

We propose a novel integrated class-sensitive and explainable framework for ICU risk management as detailed in the “Methods” section. We compare our proposed XMI-ICU gradient-boosted model to standard supervised learning methods. For some of the methods like support vector machines (SVMs), we have standardised features. All features listed in the supplementary section on data processing were used for the eICU test results while only the most important (top 8) features identified by Shapley values analysis were used for external validation on MIMIC-IV. APACHE IV score was not used as a feature in these models, albeit experiments doing so are included in the Supplementary Materials for those curious. The first set of results in Table 1 concerns the prediction of mortality in MI patients with several hours prior to the event. It is clear that XMI-ICU maintains superior performance across all metrics for a priori prediction beating state-of-the-art tabular deep learning models. For AUROC and average precision, we evaluated the model at the default risk threshold in the results presented in the tables. The results can also be seen in Fig. 1 which highlights the superior performance of XMI-ICU compared to alternative supervised learning models as measured by both AUROC and average precision.

**Table 1:** eICU test prediction results with 95 % confidence intervals for mortality prediction 6 h in advance.

	AUROC	Accuracy*	Avg precision	*p*-value
XMI-ICU	92.0 [91.1, 92.3]	82.3 [79.9, 83.5]	68.8 [65.5, 70.0]	–
Random Forest	90.6 [88.3, 91.0]	78.2 [77.7, 79.0]	64.4 [62.8, 65.1]	0.048
Logistic regression	89.6 [88.8, 90.4]	73.5 [72.4, 75.0]	61.5 [60.2, 63.0]	0.012
SVM	89.3 [88.5, 89.6]	77.0 [76.2, 77.3]	58.1 [57.2, 58.3]	0.023
GRU	89.1 [88.4, 89.8]	78.5 [77.6, 79.2]	65.0 [63.9, 65.9]	0.004
LSTM	88.6 [87.9, 89.3]	78.1 [77.0, 78.9]	64.6 [63.4, 65.5]	0.002
SVM (linear)	87.7 [86.6, 88.0]	78.8 [77.3, 79.5]	63.8 [62.9, 64.1]	0.002
NODE	85.4 [84.7, 87.0]	67.6 [66.1, 68.9]	62.3 [61.0, 63.8]	$<\!0.001$
TabNet	84.1 [83.9, 84.3]	77.0 [76.2, 77.4]	60.7 [59.1, 62.0]	$<\!0.001$
TabNet (pretrained)	82.2 [81.3, 82.7]	76.0 [75.7, 76.5]	64.1 [62.9, 65.1]	$<\!0.001$
XMI-ICU (static)	80.1 [78.9, 82.2]	70.6 [68.8, 72.0]	61.1 [59.2, 62.5]	$<\!0.001$
LDA	78.7 [77.2, 79.9]	51.0 [50.9, 51.2]	29.3 [29.1, 29.4]	$<\!0.001$

Statistical significance was assessed with DeLong (AUROC) and McNemar (accuracy); all *p*-values are relative to XMI-ICU. XMI-ICU (static) is a model trained only on static features. *Balanced accuracy.

After the XMI-ICU model was evaluated at 6-hour prediction prior to death, we extended to a more dynamic prediction evaluation by adapting the framework to arbitrarily predict the events of death at any time prior, and the framework will automatically extract, preprocess, standardise existing measurements, optimise respective hyperparameters, and deploy the XGBoost model for test prediction. The results for XMI-ICU evaluated at 6, 12, 18, and 24-h prediction for mortality in the held-out test set of eICU can be seen in Table 2 and they continue to show reliable predictive performance across the different time windows. The table also includes the results for APACHE-IV as a matter of comparison for 24 hour prediction of mortality.

**Table 2:** XMI-ICU test prediction results with 95% confidence intervals for mortality prediction across different time horizons.

	Test AUROC	Acc*	AP	p-value
eICU (All)				
6 hours	92.0 [91.1, 92.3]	82.3 [79.9, 83.5]	68.8 [65.2, 70.0]	-
12 hours	89.9 [88.7, 90.9]	81.9 [79.3, 82.1]	65.8 [62.4, 67.3]	-
18 hours	89.8 [87.9, 90.1]	81.2 [77.6, 81.5]	65.5 [62.1, 66.9]	-
24 hours	88.2 [86.3, 88.5]	80.4 [76.8, 80.7]	63.0 [59.8, 64.4]	-
APACHE IV				
24 hours	69.9 [68.9, 71.3]	69.3 [66.5, 70.9]	31.5 [30.3, 32.0]	<0.001
eICU (Top-8)				
6 hours	86.2 [85.7, 86.7]	80.0 [79.2, 80.7]	74.7 [74.1, 75.3]	<0.001
12 hours	83.3 [82.8, 83.9]	77.0 [76.1, 77.9]	69.7 [69.0, 70.4]	<0.001
18 hours	83.1 [82.5, 83.7]	76.5 [75.5, 77.4]	65.8 [65.3, 66.5]	0.044
24 hours	81.2 [80.6, 81.7]	75.2 [74.2, 76.0]	59.2 [58.8, 60.1]	<0.001
External MIMIC-IV				
6 hours	80.0 [77.9, 82.3]	77.7 [76.2, 79.7]	73.8 [71.2, 75.0]	<0.001
12 hours	77.7 [75.6, 80.1]	75.9 [74.2, 77.8]	69.9 [67.3, 71.2]	0.031
18 hours	76.6 [74.5, 79.0]	75.1 [73.4, 76.9]	67.8 [65.4, 69.5]	0.02
24 hours	75.1 [73.0, 77.6]	74.9 [73.1, 76.7]	66.5 [64.1, 67.2]	0.042

eICU results use all available features, Top-8 eICU uses only the most important features identified by Shapley analysis, and MIMIC-IV represents external validation using the top 8 features without any training on MIMIC-IV data. P-values represent statistical significance compared to eICU (All) at the same time horizon using DeLong’s test for AUROC and McNemar’s test for accuracy. *Balanced accuracy and AP stands for Average Precision.

**Fig. 2:**
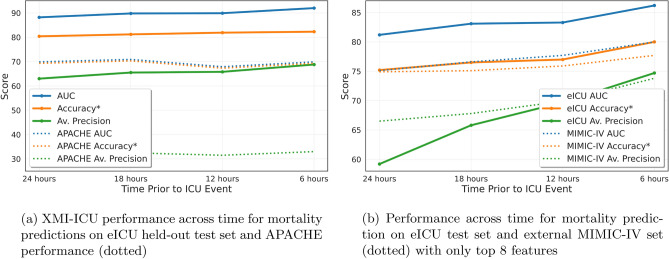
Robustness and reliability of XMI-ICU prediction performance over time in the ICU for mortality prediction (left) using all features available in eICU and as measured by a variety of metrics. The right figure contains results from the eICU held-out test set and MIMIC-IV external cohort with only the top 8 features identified by Shapley value analysis.

A plot showing the stability of predictive performance across different metrics for XMI-ICU as a function of time in the ICU prior to death can be seen in Fig. 2aindicates stable performance for mortality prediction and its superiority compared to APACHE-IV (included for times beyond just 24 h). The right figure shows the generalisation ability of the model to perform on a completely external test set, MIMIC-IV, using only the top 8 features from eICU for training. Evaluating a time-prediction model like XMI-ICU also requires showing coherent prediction across time and not just consistency of prediction accuracy and robustness. In the next set of results, we show XMI-ICU with low misclassification error across time for the same patient sample. A patient is deemed misclassified if they are predicted incorrectly at time x in advance when they have been previously predicted correctly at times >x. For example, a patient might be predicted to die at the 24 and 18-hour prediction windows correctly, but at 12 h in advance, they are predicted (incorrectly) to survive. These instabilities in prediction across time need to be measured if the model is to sustain reliable performance throughout the ICU stay. We define three patient subcohorts as illustrated at the top of Table 3, where each indicates the group of patients correctly predicted at all previous time windows except one. The bottom of Table 3 presents these results for both death and heart attack prediction, indicating the low levels of misclassification most likely indicate sensitivity to noise rather than predictive weakness.

**Fig. 3:**
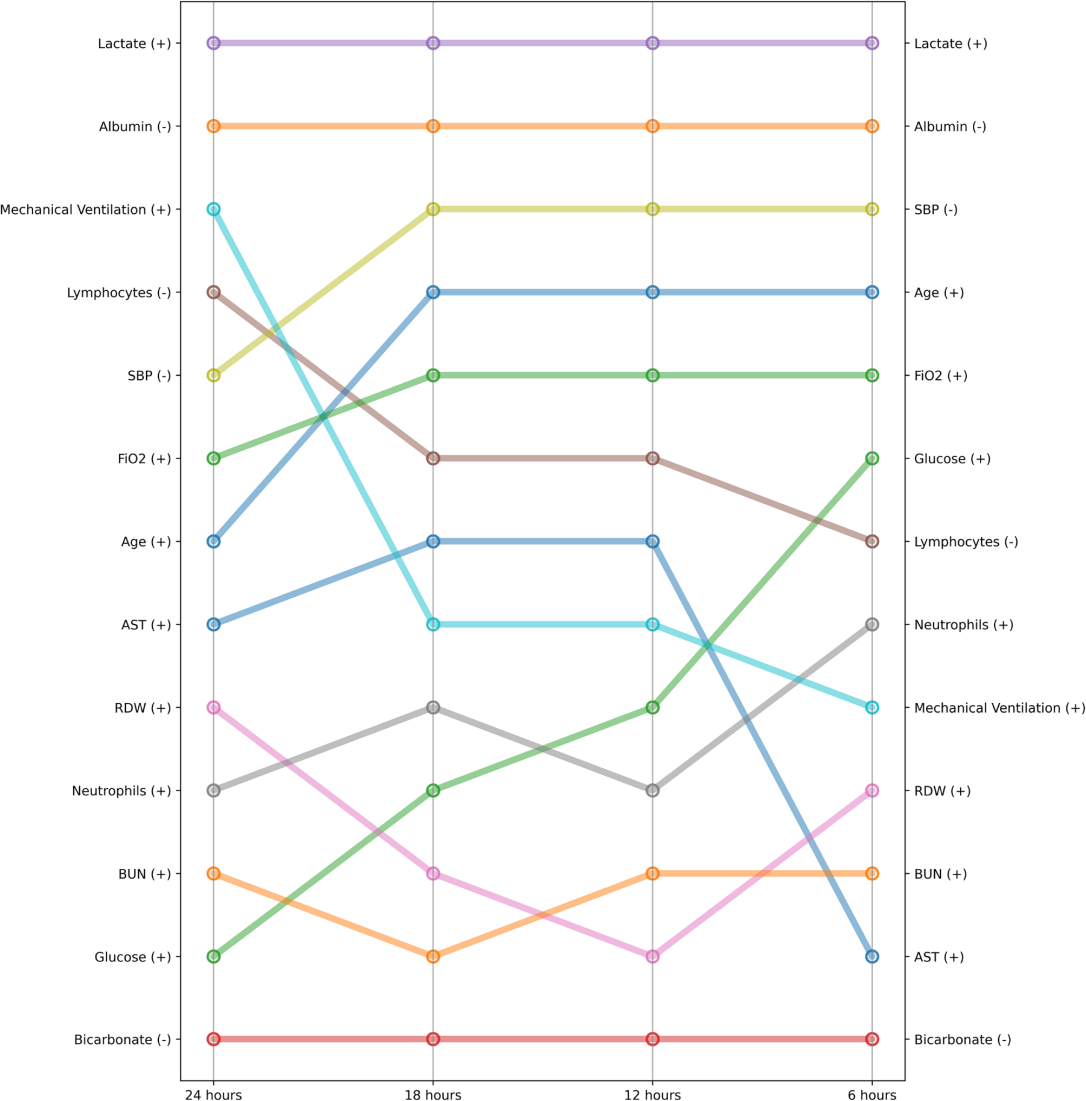
Ranking of most important features as identified by their relative SHAP values for XMI-ICU prediction of mortality varied across time during ICU stay prior to the event. The (+) and (−) signs next to each feature indicate the direction in which the feature value affects the increase in risk of death. For example, for glucose, the higher the value of glucose, the higher the predicted risk of death. Furthermore, glucose increases in relative importance for prediction the closer the patient gets to the event. For the time windows in the 6, 12, 18, 24 h intervals, the top 13 features in each of the windows are presented as extracted from eICU, thereby showcasing how the most important features for correct prediction of mortality changes through time or closer to the prediction event.

**Table 3:** TOP: Defined patient cohorts for evaluating XMI-ICU predictive robustness across time windows.

Patient cohort	24 hours	18 hours	12 hours	6 hours
${P_{1}}$	$\checkmark$	$\checkmark$	$\checkmark$	X
${P_{2}}$	$\checkmark$	$\checkmark$	X	
${P_{3}}$	$\checkmark$	X		
	${P_{3}}$	${P_{2}}$	${P_{1}}$	
eICU				
Mortality	7.9	8.2	5.5	
MIMIC-IV				
Mortality	6.4	6.3	4.7	

Each patient cohort corresponds to a grouping of patients who have been wrongly predicted at time x after being correctly predicted at all times before. BOTTOM: Misclassification rate (in percentage) is defined as the number of wrong classifications divided by total patient sample present in cohorts for 6, 12, 18, and 24 h prediction windows. A misclassification example is one where a patient is wrongly predicted in a time prediction window after being correctly predicted at previous windows.

To interpret XMI-ICU’s mortality predictions, we applied Shapley value analysis to the held-out test set and examined relative feature importance rankings. This interpretability analysis was conducted across all prediction horizons (6, 12, 18, and 24 h), with detailed results for the 6-hour prediction model presented in the Supplementary Materials (Tables S5 and S6). To validate the robustness of our interpretability findings, we conducted random perturbation tests by adding Gaussian-distributed noise features to the original feature set and evaluating whether the top-ranked variables remained stable. The introduction of noise variables produced no significant changes in the most important features identified by Shapley analysis, confirming the reliability of our interpretability findings. Complete perturbation test results are provided in the Supplementary Materials Fig. S5.

We further stratify Shapley values as a function of time in the ICU for mortality prediction. A feature ranking at each time-point corresponds to the relative importance of that feature at that point in time in the ICU stay prior to the event in question. The time graphs can be seen in Fig. 3. These values were extracted for each of the time windows, in effect converting a static interpretability method to a dynamic explainability framework that shows how at different times closer to the event (death or heart attack) different values of features and their importance change and how that is used by the model to learn underlying patterns for disease outcome prediction.

### External validation: MIMIC-IV

For external validation, we limited our analysis to the top 8 most important features identified through Shapley value analysis on the eICU dataset. First, it provides a more rigorous test of generalisability. If a model can maintain strong performance using only its most critical features on a completely independent dataset, this suggests robust identification of truly predictive clinical patterns rather than dataset-specific artifacts. Second, it addresses the clinical reality that simpler models with fewer features are more likely to be adopted in practice, as they require fewer data inputs and are easier to implement across different hospital systems with varying data collection capabilities. Third, it serves as a form of implicit regularisation, reducing the risk that our model’s performance depends on dataset-specific noise or idiosyncrasies that might not translate to other healthcare settings. We evaluated XMI-ICU on the separate and independent MIMIC-IV dataset for mortality prediction in MI patients. The features identified as most important by Shapley values analysis were used to create a new training set of the entirety of eICU and test on the entirety of MIMIC-IV cohorts only using the top 8 features whose statistical distributions for the different sets are included in Table 4. The distributions of the respective features can be seen in Supplementary Materials Figures S1, S2, and S3. XMI-ICU maintains high predictive performance across metrics when tested on this external dataset as can be seen in Table 2 without any training or tuning on it using only the top 8 features identified by Shapley value analysis from the eICU test set. The results immediately above correspond to held-out test set performance for eICU using those same 8 features.

A plot showing predictive performance across different metrics for XMI-ICU evaluated on the MIMIC-IV cohort can be seen in the bottom Fig. 2b

### Clinical risk benefit analysis

**Fig. 4:**
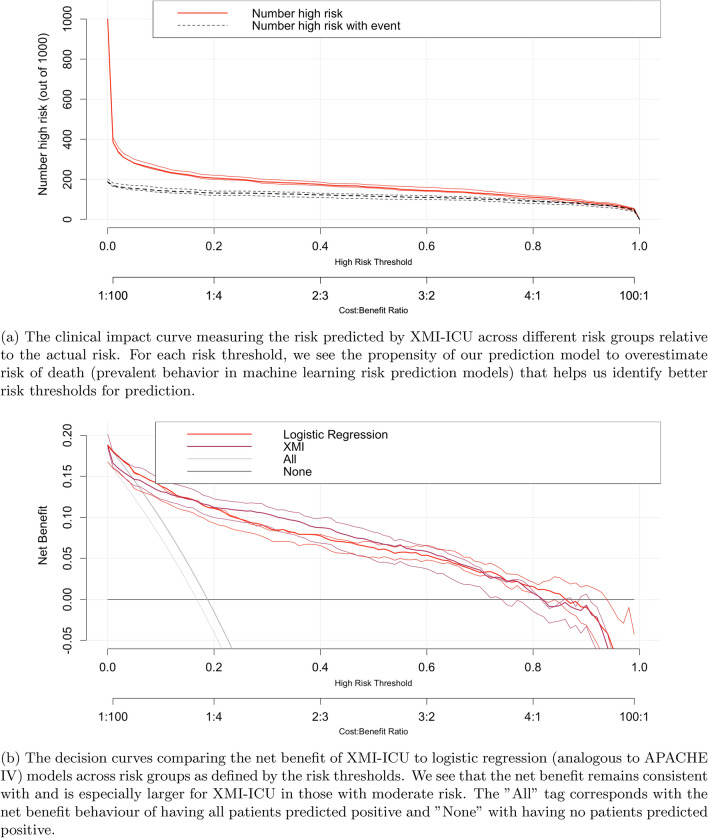
Clinical decision-making evaluation performance of XMI-ICU for mortality prediction using only the top 8 features on the entire eICU test set.

To communicate the clinical significance of the XMI-ICU model results to clinicians, we evaluated our model with clinical impact curves (Fig. 4a) and decision curve estimates (Fig. 4b) for robust risk evaluation. A 90 percent confidence interval was derived with 50 bootstrap iterations on the test set. As the clinical impact curves for mortality show, XMI-ICU consistently identifies patients at risk across different risk thresholds showing robustness to false negatives. For those at highest risk (>75%), XMI-ICU has very low tendencies for false positives or ”over-risking” in its predictions, learning to focus on those most at risk with higher specificity and sensitivity. The decision curves indicate XMI-ICU’s approximated net benefit outperforming logistic regression (underlying model used in APACHE) using only top features identified from Shapley values analysis.

To assist clinicians more readily in their decision-making process, the top features of our XMI-ICU model were used to construct a nomogram, which is included in the Supplementary Materials Figure S7 and shows a simple representation of what could be an automatic calculator for 24-h risk calculation in the ICU.

## Discussion

Our proposed XMI-ICU framework is the first comprehensive machine learning approach to mortality prediction for heart attack patients. For a condition with a significantly higher risk of death, and worse outcomes in the ICU, this presents an important question to address. XMI-ICU does not just use demographic data but also longitudinal time-series data in a pseudo-dynamic manner, coupled with a data pre-processing and Bayesian optimisation pipeline. It is robustly tested in two large heterogeneous ICU datasets, including a multi-centre ICU data source with external validation. XMI-ICU shows superior predictive performance for mortality prediction across different metrics. While our prediction time for mortality is at least 6 and up to 24 hours before death, the framework can be applied to any arbitrary time, which leaves clinicians with flexible extra time to prioritise high-risk patients and administer preventative measures. XMI-ICU presents a paradigm shift from prior approaches to mortality prediction algorithms for heart attack patients, like the GRACE, Framingham, and TIMI scores, which are simple linear risk calculators that only project long-term mortality at a fixed time point. We have also benchmarked against state-of-the-art tabular deep learning models like TabNet and NODE and have achieved superior performance with XMI-ICU. XMI-ICU provides the risk of death dynamically in the ICU for heightened responsiveness and learns relevant underlying physiological dynamics of the patient state with interpretability.

The superior performance of XMI-ICU over tabular deep learning methods can be attributed to, first, EHR data exhibits characteristics that favour tree-based models: moderate sample sizes, mixed data types combining categorical and continuous variables, and the presence of many potentially uninformative features due to the comprehensive nature of ICU monitoring. Second, the temporal aggregation strategy employed in our framework (using mean and standard deviation summaries) creates a feature space that aligns well with tree-based learning. The decision tree structure directly mirrors clinical decision-making processes, where clinicians use threshold-based reasoning (e.g., ”if lactate > 2.5 and albumin < 3.0, then higher risk”). Finally, the class imbalance in our dataset presents challenges for deep learning models that require careful architecture design and training procedures. Tree-based models naturally handle imbalanced datasets through their splitting criteria and can be easily adjusted using class weights, as demonstrated in our approach. The combination of these factors, moderate dataset size, mixed data types, noise resilience, and class imbalance handling, explains why our tree-based approach outperformed more complex deep learning alternatives, consistent with recent findings that tree-based models remain superior for many tabular prediction tasks^23^.

XMI-ICU also beats the existing prediction tool in use across ICUs in the United States, APACHE IV, by 18.3% in test AUROC and 11.1% in test accuracy at 24-hour prediction. While the APACHE IV score is calculated at admission, and thus is not the optimal comparison algorithm, it does still provide 24-hour mortality prediction, which is the time window we compared our results from. XMI-ICU requires milliseconds to be deployed once trained, which also only takes a couple of seconds, allowing for rapid response times in the ICU. Additionally, as Fig. 2ashows, XMI-ICU maintains stable performance across all metrics during the 24 h of ICU stay prior to death for MI patients. The model also successfully performs mortality prediction across different prediction time-windows in an external patient cohort obtained from MIMIC-IV without any training on the dataset using only the 8 most important features identified by Shapley values analysis on eICU as seen in Fig. 2b. The maintained performance across both eICU (using top 8 features) and MIMIC-IV external validation suggests that these features capture fundamental physiological signals associated with mortality risk in MI patients that transcend specific hospital systems and data collection protocols. This finding has important implications for clinical translation, as it suggests that effective mortality prediction can be achieved with a focused set of readily available clinical measurements, potentially facilitating broader adoption across diverse healthcare settings. The drop in predictive performance with MIMIC-IV is expected as we now use only 8 features without any training on the MIMIC-IV dataset itself. Table III also demonstrates this for the same 8 features in eICU where a decrease in performance is observed because only the few most predictive features are used. Despite this challenge, XMI-ICU maintains relatively high external predictive performance.

The added interpretability provides clinical risk factor importance, which can aid physicians in both relying on the model and investigating what aspects of physiological measurements are more informative at different times during the ICU stay. Our time-resolved Shapley value analysis represents an extension beyond traditional model interpretability, tracking feature importance across multiple prediction horizons to provide clinicians with temporal insights into risk factor evolution during ICU stay. This approach reveals that optimal clinical monitoring strategies should adapt based on prediction timeline, focusing on baseline characteristics and comorbidities for longer-term risk assessment while emphasising acute physiological measurements for short-term mortality prediction. For mortality prediction, we observe that as patients approach the highest risk of death, mechanical ventilation status drops in importance compared to blood measurements like higher lactate, lower albumin, and systolic blood pressure. Hyperlactatemia is highly associated with in-hospital mortality in relatively small and isolated heterogeneous ICU populations^29^, and our findings on a much larger multi-centre patient cohort provide predictive evidence supporting this association. Previous research has established that lower serum albumin levels are good predictors of higher risk of death in ICU patients with sepsis and COVID-19, while our work suggests a similar predictive pattern for myocardial infarction patients^30,31^. While this remains a matter of ongoing debate in medical sciences, lower albumin levels may serve as a marker of persistent arterial injury and progression of atherosclerosis and thrombosis, with prolonged low albumin levels indicating higher risk of further acute myocardial injury, making it useful for tracking MI risk as our results suggest^32,33^. Such dynamic interpretability could inform adaptive monitoring protocols and help prioritise interventions based on temporal risk patterns, providing actionable clinical insights beyond static feature importance rankings.

Prior work has shed light on the hypothesis that hypotension, as measured by the lowering of systolic blood pressure, can be an indicator of higher risks of death in ICU patients, specifically those with acute kidney injury^34^. Some have suggested that myocardial injury is also more likely in cases of lower SBP values, but here we provide an early indication of the high prediction value of lower SBP levels for heart attack in the ICU^35^. The sudden rise in high glucose levels and variability (as captured by our standard deviation measure for blood glucose) being strong predictors of mortality have been confirmed with several retrospective cohort studies in the ICU^36,37^. Work on prioritising those patients with such measurements and controlling for blood glucose and albumin can more easily be extended to preventive care for MI patients as well.

Potential limitations of the study are relatively small patient cohorts extracted from large general ICU populations. A targeted dataset consisting of only those patients with a confirmed primary diagnosis of myocardial infarction in their records can allow a deeper analysis of this population. Further work can develop larger or more complex models that can exploit the longitudinal, less sparse information if a more comprehensive data source with heart attack patient records becomes available. Comparing the framework to existing deep learning time-series models that tend to be costly and complex, our XMI-ICU framework, with its simple embedded gradient-boosted model robust against class imbalance and with dynamic feature extraction maintains prediction fidelity at varying time points while being faster, more interpretable, and less environmentally and financially costly to train and deploy. The XMI-ICU dynamic framework also offers an alternative to the rush in clinical machine learning in applying costly and less interpretable deep learning models to these types of problems, while still providing a dynamic prediction framework. Another limitation of our current approach is the relatively simple temporal feature extraction strategy. While mean and standard deviation provide robust and interpretable summaries of physiological measurements within time windows, they may not capture all relevant temporal patterns that could improve predictive performance. More sophisticated temporal features could include the min-max range, the temporal slope to identify trends in physiological parameters (improving, deteriorating, or stable), and measures of temporal autocorrelation to assess the persistence of physiological states. These additional features could potentially capture subtle temporal dynamics that our current approach might miss, particularly for patients with complex physiological trajectories. Future work could integrate transformer-based architectures, as recent studies demonstrate that transformers predict patient outcomes efficiently through training-time parallelism while their attention weights yield clinically interpretable insights into the learned dynamics^38–40^. We therefore view transformer models as a natural extension of our pseudo-dynamic framework.

In conclusion, we developed a highly predictive machine learning framework that trains on time-series ICU ward data without requiring complex deep learning models. Instead, it relies on dynamic feature extraction and takes advantage of the predictive power of static models like XGBoost, which outperformed other models, including state-of-the-art tabular deep learning. The framework offers time-resolved interpretability that allows tracking changes in vital signs and blood measurement importance across the ICU stay for heart attack patients, whose conclusions seek to provide medical insight. The framework could be integrated into ICU systems to predict negative outcomes in heart attack patients with real-time patient measurements.

## Methods

### Study design and population

The cohort data used in this study is the eICU Collaborative Research Database and the Medical Information Mart for Intensive Care (MIMIC-IV v. 2.0, July 2022), public ICU databases available upon request and fulfilment of ethical training^41,42^. The eICU database was processed using postgreSQL and the *pandas* package. eICU is a multi-centre ICU database with over 200,859 patient unit encounters for 139,367 unique patients admitted between 2014 and 2015 to one of 335 ICUs at 208 hospitals located throughout the United States^41^. The database is de-identified and includes vital sign measurements, demographic data, and diagnosis information. For a full list of features used in our study, please consult the relevant tables in the Supplementary Materials (Tables S1, S2, S3, S4).

We define our myocardial infarction cohort as patients with confirmed MI diagnosis, including both STEMI and NSTEMI presentations, as well as acute coronary syndrome patients with confirmed myocardial damage. Our inclusion criteria specifically encompass the diagnosis strings and respective ICD-10 codes described in the Supplementary Material (Table S1), which include: cardiovascular chest pain with acute coronary syndrome, acute myocardial infarction with and without ST elevation, post-PTCA myocardial infarction, and various anatomical MI locations (inferior, non-Q wave). We excluded patients with unstable angina without confirmed myocardial damage, ensuring our cohort represents true myocardial infarction cases rather than broader acute coronary syndrome presentations without tissue necrosis.

**Fig. 5:**
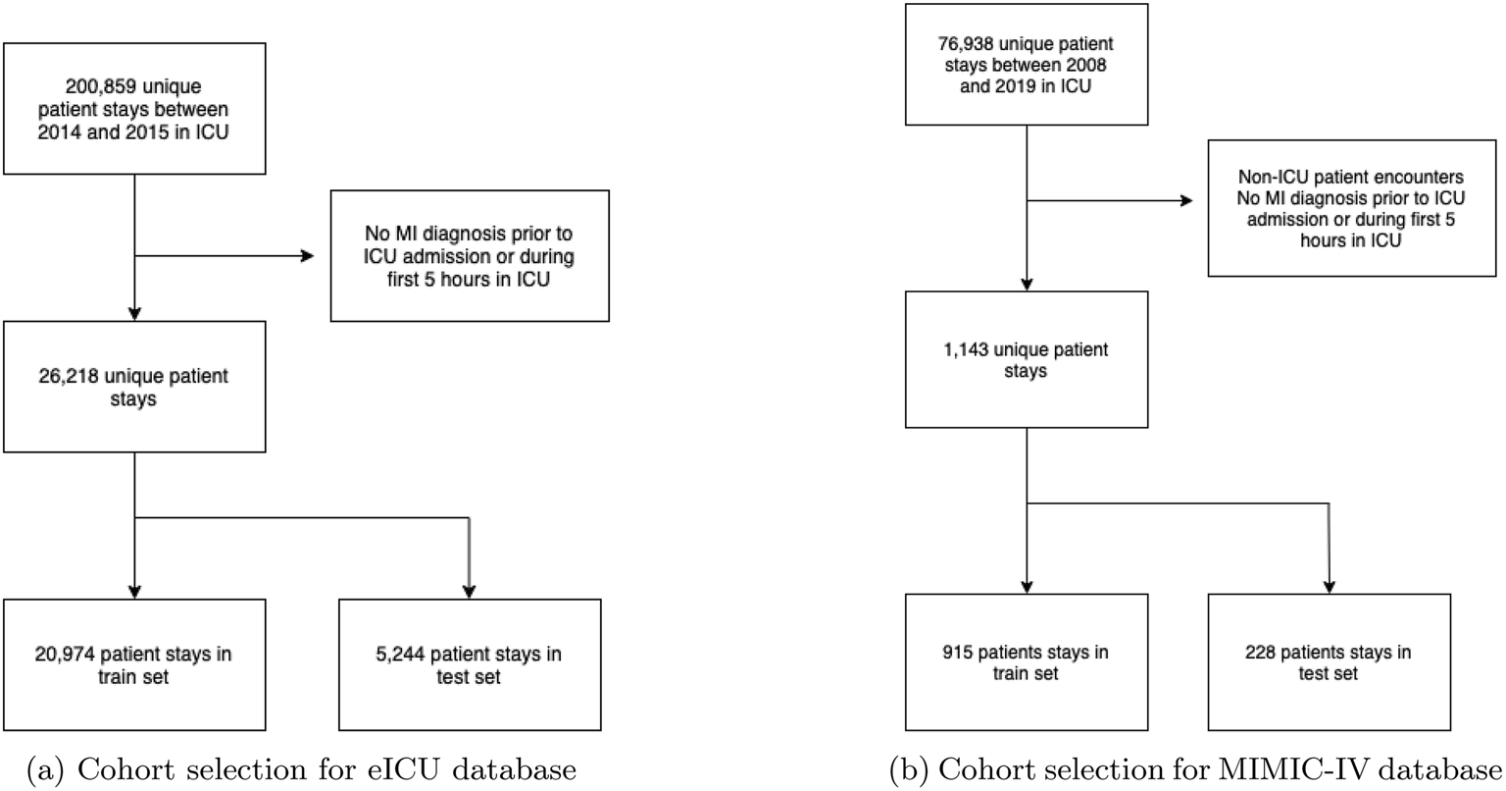
MI patient cohort selection. The exclusion criteria were listed here as they were implemented in PostgreSQL and Pandas. The final exclusion criteria are to extract the relevant subcohort at the end which is MI admitted patients to the ICU.

We based this study on the data preprocessing workflow used in^43^, but adapted it to our problem accordingly. Our inclusion criteria were patients of age>18 and <89 years with an ICU length of stay of at least 5 hours to remove transient patients. We considered only patients whose primary cause of admission was myocardial infarction (or similar as relevant) as detailed in Table S1 in the Supplementary listing the exact diagnoses. We also included those with at least one recorded observation and excluded those without any laboratory measurements. Patients on respiratory support had a separate set of measurements which we included with a mechanical ventilation tag feature for this patient subgroup. We included variables present in at least 12.5% of patient stays, or 25% for lab variables due to their relative sparsity. We then removed those patients without any diagnosis information after 5 h of stay because they might be inactive ICU patients logged for longer than was the case. A similar approach was taken by^44^. Our final subcohort consisted of 26,218 patients. We extracted diagnoses entered less than 5 hours after entering the ICU and diagnoses prior to admission as starting diagnosis or first diagnosis. A flowchart of the patients cohort selection can be seen in Fig. 5. The minimum length of stay changes depending on what model prediction time one enters into the framework. For example, evaluating the model for 6-hour or 24-hour prediction time means that the minimum length of stay for those patients would have to be 6 or 24 h for there to be existing measurements for analysis.

The eICU database as well as many of the ICUs in the United States use the APACHE IV system for mortality risk prediction. The Acute Physiology, Age, and Chronic Health Evaluation (APACHE) IV system is a tool used to risk-adjust ICU patients which provides estimates of the probability that a patient dies given data from the first 24 h^45^. The APACHE IV score is the result of a multivariate logistic regression which uses demographic, laboratory measurement, and diagnosis data to make a mortality risk assessment. It is the standard benchmark for mortality prediction tools in the ICU. Here we will use it both as a feature and a benchmark as it was highly important for us to evaluate the score’s feature importance downstream in our models. We will provide XMI-ICU prediction performance for 24 hours which is the most directly comparable to APACHE-IV. APACHE-IV is only present in the eICU dataset and we acknowledge that the score determines hospital mortality risk for all ICU patients and not just heart attack patients. Nevertheless, it provides a standard and consistent benchmark for mortality prediction in the ICU. Once we have the defined outcome as MI, we are left with 26,218 samples that have had a diagnosis of MI prior to admission to the ICU with 3139 (12.0%) having died during their stay.

We externally validated our model on Medical Information Mart for Intensive Care (MIMIC-IV v. 2.0, July 2022) which includes discharge information for over 15,000 additional ICU patients compared to the previous release^42^. Similar to eICU, MIMIC-IV is a de-identified and real-world intensive care database using data from the Beth Israel Deaconess Medical Center for the years 2008–2019. We use similar cohort selection criteria as for eICU only extracting confirmed MI diagnosis patients as illustrated in Fig. 5 and label definition as in eICU resulting in 1143 unique patient ICU stays with confirmed MI out of 76,938. 131, or 11.5%, have died during their stay. Regarding the missingness of variables, we mimic the steps taken for eICU processing. Since these datasets have different predictors and feature availability, we use the most important predictors from the eICU test set as identified by our interpretability framework to extract only those predictors from MIMIC-IV. This approach serves multiple purposes: (1) it ensures feature availability across both datasets, as not all eICU features are present in MIMIC-IV due to different data collection protocols and hospital systems, (2) it provides a stringent test of model generalisability by evaluating whether the most critical predictive features identified in one dataset maintain their predictive power in an independent cohort, (3) it reduces the risk of overfitting to dataset-specific noise by focusing on the most robust predictive signals, and (4) it demonstrates clinical practicality by showing that a parsimonious model using only key features can maintain strong performance across different healthcare systems. These top 8 features (Table 4) from eICU are also extracted from MIMIC-IV, which is wholly used as a test set (as is done in external validation), and the model is never trained on any MIMIC-IV data. These top 8 features (Table 4) from eICU are also extracted from MIMIC-IV, which is used entirely as a test set (as is done in external validation), and the model is never trained on any MIMIC-IV data.

The study follows the TRIPOD checklist which can be found on page 2 of the Supplementary.

Our outcome of interest for prognosis is general mortality in ICU as registered in the discharge information for each patient. The data processing of time-series and static variables was completed in Python. Patient cohort characteristics can be seen in Table 4.

**Table 4:** Summary of demographics and variables used for external validation across training and testing datasets. MIMIC-IV has been used separately as an external validation source with the entire dataset used as a test set.

Attributes	eICU (N = 26,218)		MIMIC-IV (N = 1,143)
	Train (N = 20,974)	Test (N = 5,244)	
Age (mean ± SD)	66.8 (± 12.7)	67.2 (± 12.4)	68.1 (± 13.2)
Sex (male)	13,369 (63.7%)	3,385 (64.5%)	585 (51.9%)
Ethnicity (male)	13,369 (63.7%)	3,385 (64.5%)	585 (51.9%)
LoS (days)	4.1 (± 2.7)	4.0 (± 2.3)	3.7 (± 2.9)
Lactate	2.9 (± 2.8)	2.5 (± 2.3)	2.0 (± 1.5)
SBP	120.2 (± 17.9)	120.0 (± 16.3)	126.3 (± 18.8)
Glucose	150.4 (± 61.7)	147.3 (± 56.7)	136.5 (± 49.3)
WBC	15.5 (± 10.5)	15.1 (± 9.3)	10.6 (± 7.4)
RDW	15.1 (± 2.2)	15.0 (± 2.0)	14.4 (± 2.1)
Urea nitrogen	27.4 (± 19.5)	22.8 (± 13.4)	22.8 (± 17.0)
Bicarbonate	24.7 (± 4.2)	24.8 (± 4.4)	23.3 (± 3.1)
Mortality (dead)	2,511 (12.0%)	628 (12.0%)	105 (11.5%)

### Machine learning methods

Following the extraction of patients, we split the dataset into training and testing (20%) with the test set being used as a hold-out for reporting only the final results. The training set was used for hyperparameter tuning of different machine learning and deep learning models using Bayesian optimisation (to help reduce the overall computational costs of the framework). The next step in the framework is to pad the missing measurements for the time windows using imputation with Multivariate Imputation by Chained Equation (MICE) and for feature standardisation or normalisation where necessary to avoid any data leakage either inside the validation folds or, in the end, the held-out test set with the parameters extracted only on the training set or the training folds respectively^46^. Instead of using resampling techniques like SMOTE which can incur bias, we use inverse class-weighting in the training phase of the models which successfully allows it to generalise to an imbalanced prediction scenario^47^. Once the models were optimised, they were compared using their generalisation capability as evidenced by the test set metrics. The metrics used included Area-Under-Receiver-Operating-Curve (AUROC or AUROC), Sensitivity, and Average Precision (AP) as they most completely capture the predictive performance of these binary classifiers even in cases of class imbalance. Details on how the metrics are calculated can be seen in the Supplementary Materials.

The XMI-ICU framework uses an extreme gradient-boosting approach with rolling time windows to extract the relevant features at defined times. This is a low-cost, time-efficient, imbalance-robust, and interpretable framework of dynamically predicting outcomes without relying on complex transformer models for time-series analysis. The benefits of transforming a time-series dynamic prediction into n-time-window static predictions for each time point are highlighted in the Supplementary Figure S4.

For time-series measurements, we leverage the advantage of gradient boosting performance previously established on tabular data. We extracted summary features like the mean and standard deviation (to preserve the units necessary for later interpretability) for each patient stay for each feature for each time window. A time window is defined as the time since admission to the x -(minus) prediction horizon time prior to the event of interest, where x is the time of the event. For example, for 24-hour prediction, we use all measurements since admission into the ICU stay until 24 hours prior to the event to model a prediction scenario. Using inverse class-weighting and a sliding time window, this framework enables users or clinicians to obtain estimates for risk of death at varying times in the future through dynamic feature extraction. For each time window, features are defined with means and standard deviations. For our experiments, the time-varying performance during different time window trials of 6, 12, 18, and 24 h prior to death was evaluated, and Shapley values were used to ascertain the interpretability of the model for each of the time windows and comment on the clinical significance of risk factors (also evaluated over time periods)^48^. As ICU monitoring generates measurements at varying frequencies depending on clinical protocols, patient acuity, and measurement type, we implemented several strategies. All measurements within each time window were included in the summary statistics calculation, regardless of their temporal distribution, ensuring that no clinical information was discarded due to irregular sampling patterns. For patients with very few measurements (fewer than 3 observations) within a time window for a given feature, we applied forward-fill imputation from the most recent available value to ensure statistical stability of the summary measures. The standard deviation calculation serves a dual purpose: it captures both the physiological variability of the parameter and implicitly reflects the measurement frequency and consistency within the window. Higher standard deviations may indicate either true physiological instability or irregular measurement patterns, both of which are clinically relevant. Missing measurements were handled using Multivariate Imputation by Chained Equations (MICE) applied after the time window summarisation, ensuring that the temporal structure was preserved before imputation.

**Fig. 6:**
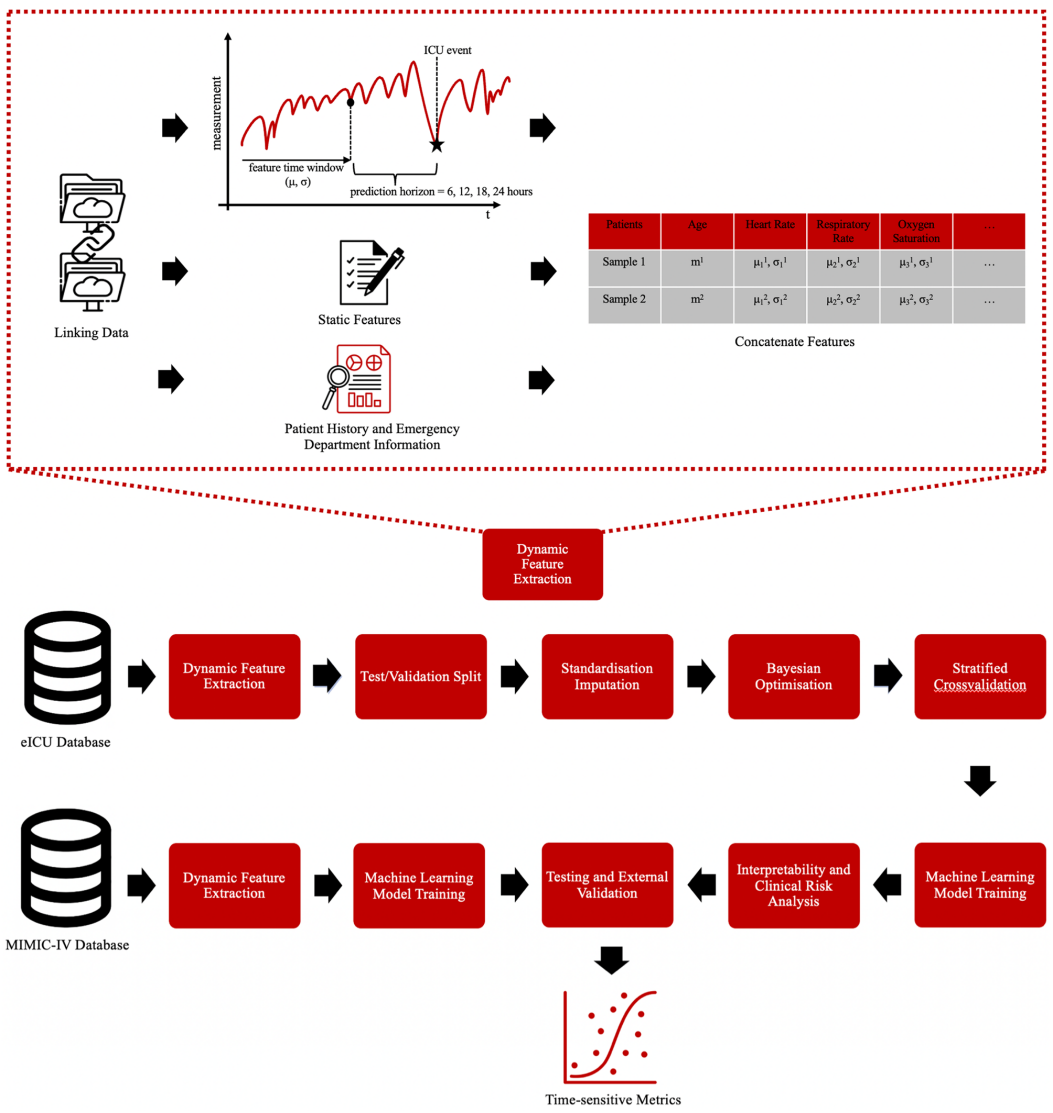
Proposed XMI-ICU framework for dynamic mortality prediction in heart attack ICU Patients. The top part of the figure shows the dynamic feature extraction that links hospital-wide data, including pre-admission information, ICU stay measurements, and emergency department variables. The sliding time windows change depending on the required prediction time, and the time-series values are summarised using mean and standard deviations. For example, for 24-hour prediction, we use all time-series measurements from the time of admission until 24 hours prior to the event as our feature time window to be summarised. The measurements are then concatenated with anamnesis, emergency department, and static variables to construct the feature matrix. The bottom half of the figure showcases the framework and how the dynamic feature extraction integrates with other components.

A flowchart visualising the proposed framework for mortality prediction in MI patients can be seen in Fig. 6. When making predictions at any time of interest, ie. 6 or 24 h, our framework only extracts the measurements collected 6 or 24 h before the event to be used as features in training the model. Thus, we guarantee that the features we use in any of our predictions are always observed with a selected time gap prior to the outcome. As Fig. 6 illustrates in the bottom half of the figure, the ICU event (death) is always a prediction horizon (at least 6 hours) away from the last considered measurement.

As far as the methodology of the deep learning models applied, TabNet and NODE, is concerned, more information can be found in the Supplementary Materials. To benchmark classical sequential models we implemented two bidirectional recurrent architectures, a two–layer Long Short–Term Memory (LSTM) network and a two–layer Gated Recurrent Unit (GRU) network. Irregular vital–sign sequences were binned onto an hourly grid, with forward filling for gaps $\le 3$ h and masking otherwise. The best configuration of hyperparameters is reported in Supplementary Section 3.

We used the same training, stratified (5-fold cross-) validation, and held-out test sets across all of our models with the hyperparameter search space included in the Supplementary Material with the selected best-performing parameters in bold. Our analysis was completed in Python 3.8 using Jupyter, pandas, numpy, SHAP, the original TabNet implementation, and the extension of the NODE implementation in PyTorch Tabular with some modifications. Decision curves, clinical risk calculations, and nomograms were computed and plotted in R. The trained model on the top features was saved in a weights file and can be loaded using the XGBoost library package to be tested as needed.

Statistical significance between model performances was assessed using DeLong’s test for comparing AUROCs and McNemar’s test for comparing accuracies, with p < 0.05 considered statistically significant^49,50^. Confidence intervals for test set metrics were calculated using bootstrap resampling with 1000 iterations.

### Interpretability

We introduce ”time-resolved Shapley value analysis” that extends traditional SHAP interpretability^51^ to our pseudo-dynamic framework. While conventional Shapley analysis provides feature importance for a single model, our approach computes Shapley values independently for each temporal prediction model (6, 12, 18, and 24 h before the event), then tracks how feature importance rankings evolve across these time windows. This temporal trajectory of feature importance reveals dynamic clinical insights, for instance, how laboratory measurements like lactate gain importance relative to demographic factors as prediction horizons narrow, reflecting the clinical reality that acute physiological derangements become more predictive of imminent mortality than baseline characteristics^52^. By transforming static interpretability into dynamic clinical insight, this time-resolved approach provides actionable information about which physiological measurements warrant closest attention at different stages of critical illness, potentially informing adaptive monitoring protocols and intervention timing strategies^53^.

### Clinical risk analysis

To provide additional analysis of the model, we used clinical impact and decision curves in estimating the performance of the model at various risk thresholds. While decision curves are mostly used in cases of intervention effect on prognosis, they can also be used to diagnose the performance of predictive models albeit their adoption in machine learning has not been widespread, possibly due to applied machine learning work in healthcare being based more on advances in computer science rather than clinical significance. Decision curves account for both the benefits of higher risk estimation and the costs of overestimating risk to a patient who cannot benefit from the prediction. They are suggested to be an improvement over measures of performance such as AUROC. The intuition behind them is if a risk model tends to identify cases as high risk without falsely identifying too many negatives as high risk, then the net benefit of the risk model to the population will be positive^54^. A mathematical representation can be seen in the equation below:

1 $$\begin{aligned} N B_R=T P R_R P-\frac{R}{1-R} F P R_R(1-P) \end{aligned}$$

Where NB is the net benefit, TPR and FPR are the positive rates, and P is the prevalence and R is the risk threshold respectively. They allow us to evaluate the models across a range of risk thresholds and observe the tendencies of the model to overestimate risk. A clinical impact curve is simpler in that it displays the estimated number of people declared high-risk for each risk threshold and visually displays the proportion of cases (true positives)^55^.

## Supplementary Information

Supplementary Information.

## Data Availability

The data is available from public access requests for eICU and MIMIC-IV. Accessing the data requires ethics module training and certification.

## Code availability

The code to implement the data processing, analyses, as well as to conduct all of the experiments, can be found in easily readable notebooks at the following repository: https://github.com/anony10subm/XMI-ICU.
