# Supplementary material for: Explainable machine learning for predicting ICU mortality in myocardial infarction patients using pseudo-dynamic data
Source: Sci Rep. 2025 Jul 31;15:27887. doi: 10.1038/s41598-025-13299-3 (PMC12311211; doi:10.1038/s41598-025-13299-3)
Supplement: Supplementary file 1 — Supplementary Information. [file 41598_2025_13299_MOESM1_ESM.pdf]

1 Libraries and Packages

Statistical and clinical analyses were performed using R (version 3.6.2, R Foundation for Statistical Computing, Vienna, Austria) with packages including binom, Epi, ggplot2, lme4, sjstats, tableone, and tidyverse. Machine learning components were coded using Python (version 3.8.0) with packages including imblearn, matplotlib, skopt, xgboost, seaborn, shap, pandas, numpy, and sklearn.

The paper follows the TRIPOD guidelines as listed in the document attached.

TRIPOD Checklist: Prediction Model Development and Validation

Section/Topic	Item		Checklist Item	Page
**Title and abstract**				
Title	1	D;V	Identify the study as developing and/or validating a multivariable prediction model, the target population, and the outcome to be predicted.	1
Abstract	2	D;V	Provide a summary of objectives, study design, setting, participants, sample size, predictors, outcome, statistical analysis, results, and conclusions.	1
**Introduction**				
Background and objectives	3a	D;V	Explain the medical context (including whether diagnostic or prognostic) and rationale for developing or validating the multivariable prediction model, including references to existing models.	2, 3
	3b	D;V	Specify the objectives, including whether the study describes the development or validation of the model or both.	3
**Methods**				
Source of data	4a	D;V	Describe the study design or source of data (e.g., randomized trial, cohort, or registry data), separately for the development and validation data sets, if applicable.	4
	4b	D;V	Specify the key study dates, including start of accrual; end of accrual; and, if applicable, end of follow-up.	4, 6
Participants	5a	D;V	Specify key elements of the study setting (e.g., primary care, secondary care, general population) including number and location of centres.	4, 6
	5b	D;V	Describe eligibility criteria for participants.	4, 5
	5c	D;V	Give details of treatments received, if relevant.	4
Outcome	6a	D;V	Clearly define the outcome that is predicted by the prediction model, including how and when assessed.	6
	6b	D;V	Report any actions to blind assessment of the outcome to be predicted.	6
Predictors	7a	D;V	Clearly define all predictors used in developing or validating the multivariable prediction model, including how and when they were measured.	4, Supplement
	7b	D;V	Report any actions to blind assessment of predictors for the outcome and other predictors.	4
Sample size	8	D;V	Explain how the study size was arrived at.	4-6
Missing data	9	D;V	Describe how missing data were handled (e.g., complete-case analysis, single imputation, multiple imputation) with details of any imputation method.	6
Statistical analysis methods	10a	D	Describe how predictors were handled in the analyses.	4-6
	10b	D	Specify type of model, all model-building procedures (including any predictor selection), and method for internal validation.	6-8
	10c	V	For validation, describe how the predictions were calculated.	6
	10d	D;V	Specify all measures used to assess model performance and, if relevant, to compare multiple models.	7
	10e	V	Describe any model updating (e.g., recalibration) arising from the validation, if done.	6
Risk groups	11	D;V	Provide details on how risk groups were created, if done.	
Development vs. validation	12	V	For validation, identify any differences from the development data in setting, eligibility criteria, outcome, and predictors.	6
**Results**				
Participants	13a	D;V	Describe the flow of participants through the study, including the number of participants with and without the outcome and, if applicable, a summary of the follow-up time. A diagram may be helpful.	4,5
	13b	D;V	Describe the characteristics of the participants (basic demographics, clinical features, available predictors), including the number of participants with missing data for predictors and outcome.	4-6, Supplement
	13c	V	For validation, show a comparison with the development data of the distribution of important variables (demographics, predictors and outcome).	7
Model development	14a	D	Specify the number of participants and outcome events in each analysis.	7
	14b	D	If done, report the unadjusted association between each candidate predictor and outcome.	
Model specification	15a	D	Present the full prediction model to allow predictions for individuals (i.e., all regression coefficients, and model intercept or baseline survival at a given time point).	
	15b	D	Explain how to the use the prediction model.	8
Model performance	16	D;V	Report performance measures (with CIs) for the prediction model.	10, 11
Model-updating	17	V	If done, report the results from any model updating (i.e., model specification, model performance).	
**Discussion**				
Limitations	18	D;V	Discuss any limitations of the study (such as nonrepresentative sample, few events per predictor, missing data).	16
Interpretation	19a	V	For validation, discuss the results with reference to performance in the development data, and any other validation data.	15
	19b	D;V	Give an overall interpretation of the results, considering objectives, limitations, results from similar studies, and other relevant evidence.	14-16
Implications	20	D;V	Discuss the potential clinical use of the model and implications for future research.	16, 17
**Other information**				
Supplementary information	21	D;V	Provide information about the availability of supplementary resources, such as study protocol, Web calculator, and data sets.	Supplement
Funding	22	D;V	Give the source of funding and the role of the funders for the present study.	17

*Items relevant only to the development of a prediction model are denoted by D, items relating solely to a validation of a prediction model are denoted by V, and items relating to both are denoted D;V. We recommend using the TRIPOD Checklist in conjunction with the TRIPOD Explanation and Elaboration document.

Table S1: Diagnoses Taken for MI Definition

**Diagnosis String**
cardiovascular—chest pain / ASHD—acute coronary syndrome
ASHD—acute coronary syndrome—acute myocardial infarction (no ST elevation)
ASHD—acute coronary syndrome—acute myocardial infarction (with ST elevation)
ASHD—acute coronary syndrome—s/p PTCA / myocardial infarction
ASHD—coronary artery disease / myocardial infarction
ASHD—coronary artery disease—known / myocardial infarction
Acute MI location
Acute MI location—inferior
Acute MI location—non-Q
Non-operative—Diagnosis—Cardiovascular—Infarction, acute myocardial (MI)
Cardiovascular (R)—Myocardial Infarction
Cardiovascular (R)—Myocardial Infarction—MI - date unknown
Cardiovascular (R)—Myocardial Infarction—MI - remote
Cardiovascular (R)—Myocardial Infarction—MI - within 6 months

2 Data Description

We defined our outcome of interest using the most common diagnosis strings associated with the myocardial infarction diagnosis as can be seen in Table S1 below.

A detailed list of features used in the study and extracted from eICU and MIMIC-IV can be seen in Tables S2 and S3.

A comparison of distributions between top features in the prediction between the two datasets is included below for age, lactate, systolic blood pressure in Figures S1, S2, S3. It clearly shows similar scale and behaviour, stabilising the external validation results from training on eICU and testing on MIMIC-IV.

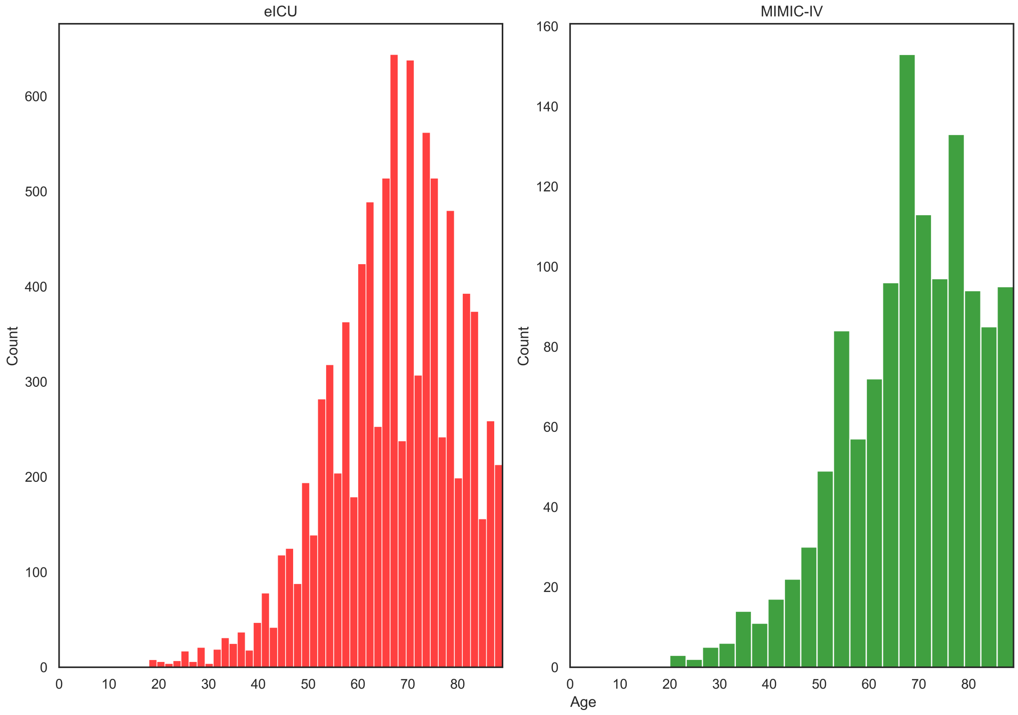

Figure S1: Age distribution between eICU and MIMIC-IV

3 Static and Tabular vs. Time-series models

As Figure S4 highlights, using compact, low-cost, and highly predictive means tested static models like XGBoost has its advantages over using complex, opaque, and slow deep learning models for raw time-series data if one is willing to invest the time learning the data and the relevant data preprocessing required.

TabNet reshapes static features using sequential splits of features with a transformer and then applies attention on these sequences at each decision step to determine the most impactful features [1]. In effect, these sequential steps are feature selections on an instance-level repeated for each sample, with a single module used for both feature selection and reasoning. A useful attribute

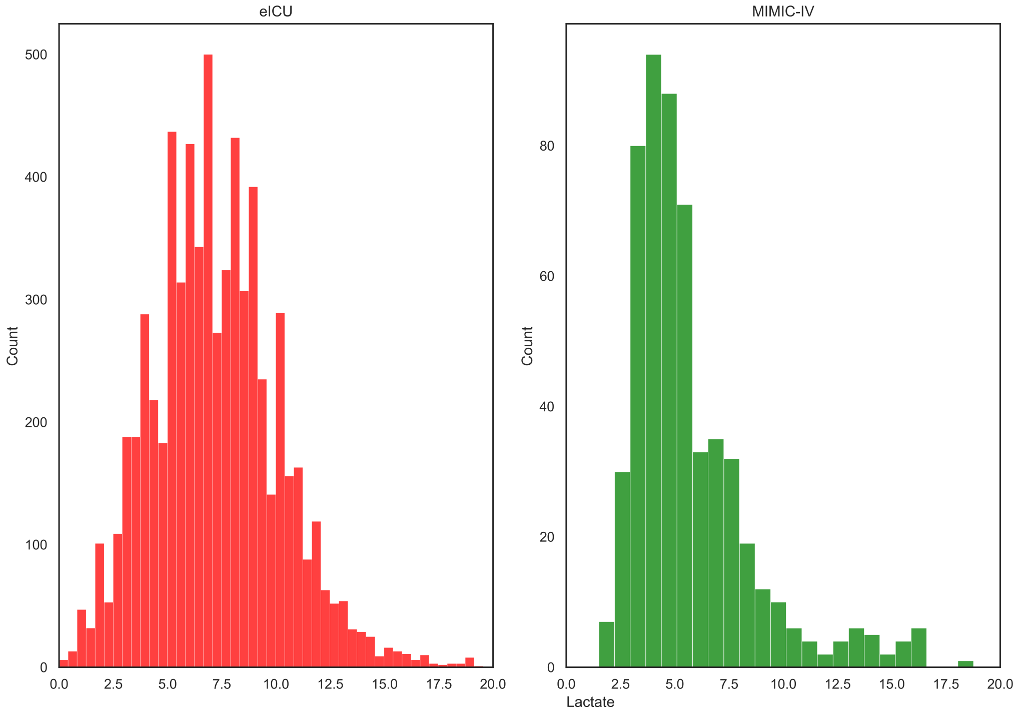

Figure S2: Lactate distribution between eICU and MIMIC-IV

of TabNet is also its self-supervised learning option, which allows one to pre-train a TabNet model on a certain fraction of features of the data before being used for training on the full model. Initial results show that this helps increase performance on small datasets (albeit larger than ours), which we will also investigate here.

NODE is another alternative to the supremacy of gradient-boosted models for tabular data which relies on similar hierarchical representation learning. In short, NODE extends the idea of gradient boosting on oblivious decision trees or decision tables and it does so by making splitting features and decision tree routing differentiable. It then adds an entmax transformation which maps a vector of real values to a discrete and sparse probability distribution. This addition allows differentiable

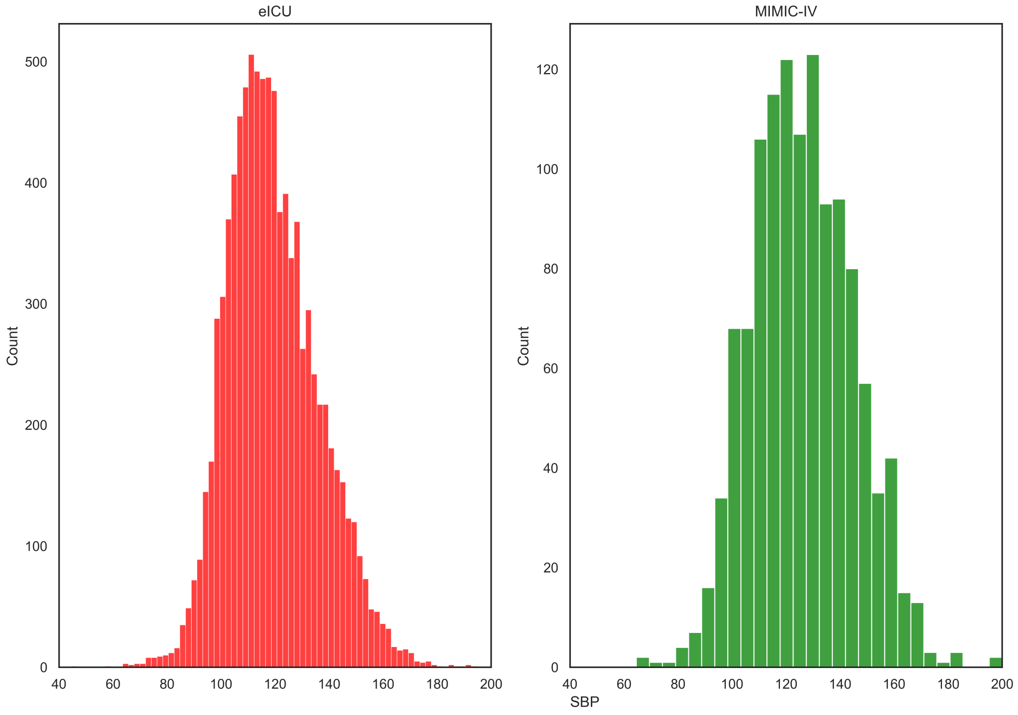

Figure S3: SBP distribution between eICU and MIMIC-IV

split decision construction in the internal tree nodes, leading to higher efficiency and resistance to overfitting [2]. The output is a sum of leaf responses scaled by the choice weights.

For the LSTM and GRU, each recurrent layer contained 128 hidden units and was followed by a 64–unit fully connected layer. We applied dropout of 0.2 and layer normalisation after each recurrent layer. Models were trained with Adam (learning rate 1×10^{-3}), mini–batch size 128, for up to 100 epochs with early stopping (patience = 10 on validation AUROC). Class imbalance was handled using inverse–frequency weighting identical to XMI–ICU. Hyper–parameters such as hidden size $\in \{64, 128, 256\}$, sequence length $\in \{12\,\text{h}, 24\,\text{h}\}$, and dropout $\in \{0.1, 0.2, 0.3\}$, were tuned by 5–fold stratified cross–validation;

4 Bayesian Optimisation Method

Bayesian optimisation relies on using a Gaussian Process (GP) defined by the property that any finite set of N points $\{\mathbf{x}_n \in \mathcal{X}\}_{n=1}^{N}$ to induce a multivariate Gaussian distribution:

$$f : \mathcal{X} \to \mathbb{R}$$

With observations $\{\mathbf{x}_n, y_n\}_{n=1}^{N}$, where $y_n \sim \mathcal{N}(f(\mathbf{x}_n), \nu)$ and ν is the variance of noise. The acquisition function is described as $a : \mathcal{X} \to \mathbb{R}^{+}$ and determines what point in $\mathcal{X}$ should be evaluated next via optimization $\mathbf{x}_{\text{next}} = \text{argmax}_{\mathbf{x}}\, a(\mathbf{x})$. The acquisition functions depend on the previous observations, as well as the GP hyperparameters. The goal is then to maximise the expected improvement (EI) over the current best and use the highest utility hyperparameter values in computing the loss.

When maximising the EI, we sample from the set of unexplored points without trying out all possible hyperparameter combinations. The algorithm can be shortly described as:

1. Given observed values $f(\mathbf{x})$, update the posterior using the GP model
2. Find $\mathbf{x}_{\text{new}}$ that maximises the EI: $\mathbf{x}_{\text{new}} = \arg\max EI(\mathbf{x})$
3. Compute the loss for the point $\mathbf{x}_{\text{new}}$

5 Metrics

The metrics used to evaluate the models include:

1. Area under receiver-operating-characteristic curve (AUROC): an ROC curve is a plot of true positives (TP) as a function of false positives (FP) where each point on the ROC curve repre-

sents a sensitivity/specificity pair corresponding to a particular decision threshold. The area under the ROC curve is a summary measure of sensitivity and specificity [3].

2. Sensitivity, the probability of a positive prediction for patients with disease (i.e. the conditional probability of correctly identifying diseased patients)

$$\frac{TP}{TP + FN}$$

3. Specificity, the probability of a negative prediction for patients without the condition

$$\frac{TN}{TN + FP}$$

4. Accuracy, ratio between correctly classified examples and the total number of cases in the dataset. In our case, can be misleading because of class imbalance where simply assigning all examples to the majority class is a way of achieving high accuracy, so instead we rely on using balanced accuracy as the average of sensitivity and specificity instead

$$\frac{Sensitivity + Specificity}{2}$$

5. Average Precision (AP) summarizes a precision-recall curve as the weighted mean of precisions achieved at each threshold, with the increase in recall from the previous threshold used as the weight:

$$\text{AP} = \sum_n (R_n - R_{n-1}) P_n$$

where P_n and R_n are the precision and recall at the nth threshold [1]. This implementation is not interpolated and is different from computing the area under the precision-recall curve with the trapezoidal rule, which uses linear interpolation and can be too optimistic. AP is then similar to using the midpoint rule for estimating the area (hence "average" precision)

6 Interpretability Methods

We use the *shap* library and built on the game-theoretic concept of treating features in the final model as players in a voting game. The method is applied on the entire test set and is based on ideas from game theory [4,5]. In short, the following equation is used to calculate the Shapley value φ for feature i:

$$\varphi_i(v) = \sum_{S \subset N \setminus \{i\}} \frac{|S|!(n - |S| - 1)!}{n!} (v(S \bigcup \{x_i\}) - v(S)) \tag{1}$$

Where features have their value calculated by taking the difference between the results of the characteristic function v on N (the set of all features) and S (the subset of N without feature i). The Shapley value of a particular feature i is then calculated by taking the average of the marginal contributions of all possible combinations.

As Figure S5 highlights the application of this method for mortality prediction. This set of results relates only to the 6-hour prediction task. We then added a Gaussian distributed feature to the feature set to evaluate the susceptibility of the top variables as identified by Shapley values changing, and we can see in Figure S5 that the interpretability provided remains robust to noise.

7 Machine Learning Methods

8 Additional Results

We also evaluated our framework for internal validation with MIMIC-IV separately ie. training and tuning on MIMIC-IV train set and testing on a held-out MIMIC-IV test set. The results are a lot

more robust than for external validation as we might expect and can be found in Figure S6.

Results of evaluating XMI specific subpopulations can be seen in Table S5.

The nomogram in Figure S7 is an example of risk calculation where one first draws a line from each parameter value to the point line for the point for that feature, then the points for all the features are added up, after which a line from the total points line is drawn vertically to determine the risk of mortality on the lower line of the nomogram as defined by a linear transformation of risk probabilities.

References

[1] Arik SÖ, Pfister T. Tabnet: Attentive interpretable tabular learning. In: Proceedings of the AAAI Conference on Artificial Intelligence. vol. 35; 2021. p. 6679-87.

[2] Popov S, Morozov S, Babenko A. Neural oblivious decision ensembles for deep learning on tabular data. arXiv preprint arXiv:190906312. 2019.

[3] Zou KH, O'Malley AJ, Mauri L. Receiver-operating characteristic analysis for evaluating diagnostic tests and predictive models. Circulation. 2007;115(5):654-7.

[4] Lundberg SM, Erion G, Chen H, DeGrave A, Prutkin JM, Nair B, et al. From local explanations to global understanding with explainable AI for trees. Nature machine intelligence. 2020;2(1):56-67.

[5] Ibrahim L, Mesinovic M, Yang KW, Eid MA. Explainable prediction of acute myocardial infarction using machine learning and shapley values. IEEE Access. 2020;8:210410-7.

Table S2: Features extracted from the eICU database. The features include demographic data collected for all patients, ICU unit-specific information like type and number of beds, hospital information like regional location and teaching status, vital signs including respiratory rate and blood pressure, and biochemical measurements including troponin and levels of potassium and protein in the blood.

**Feature**	**Type**	**Feature**	**Type**
Sex	binary	Unit Stay Type	categorical
Age	integer	Num Beds Category	categorical
Height	continuous	Region	categorical
Weight	continuous	Teaching Status	binary
Ethnicity	categorical	Physician Speciality	categorical
Unit Type	categorical	Unit Type	categorical
Unit Admit Source	categorical	Mechanical Ventilation	binary
**Time-series Variables**			
Feature	*Type*	*Feature*	*Type*
		Base Excess	continuous
-basos	continuous	FiO2	continuous
-eos	continuous	HCO3	continuous
-monos	continuous	Hct	continuous
-polys	continuous	Hgb	continuous
ALT	continuous	MCH	continuous
AST	continuous	MCHC	continuous
BUN	continuous	MCV	continuous
O2 Sat (%)	continuous	MPV	continuous
PT-INR	continuous	PT	continuous
RBC	continuous	PTT	continuous
RDW	continuous	WBC	continuous
Alkaline ph.	continuous	Albumin	continuous
Bedside Glucose	continuous	Anion Gap	continuous
Calcium	continuous	Bicarbonate	continuous
Creatinine	continuous	Glucose	continuous
Lactate	continuous	Magnesium	continuous
pH	continuous	paCO2	continuous
paO2	continuous	Phosphate	continuous
Platelets	continuous	Potassium	continuous
Sodium	continuous	Bilirubin	continuous
Protein	continuous	Troponin - I	continuous
Urinary s. Gravity	continuous	mean BP	continuous
SBP	continuous	DBP	continuous

Table S3: Features extracted from the MIMIC-IV database.

**Static Variables**			
Feature	*Type*	*Feature*	*Type*
Sex	binary	Motor Response	continuous
Age	integer	Verbal Response	continuous
Height	continuous	Ethnicity	categorical
Weight	continuous	Unit Type	categorical
Hour of Admission	integer	Admission Location	categorical
**Time-series Variables**			
Feature	*Type*	*Feature*	*Type*
Eye Response	continuous	GCS - Motor	continuous
Braden Score	continuous	GCS - Verbal	continuous
Strength L Arm	continuous	Daily Weight	continuous
Strength R Arm	continuous	ALT	continuous
Strength L Leg	continuous	AST	continuous
Strength R Leg	continuous	HCO3	continuous
Insurance	categorical	Hct	continuous
ALT	continuous	Alkaline Phosphatase	continuous
Anion Gap	continuous	AST	continuous
Base Excess	continuous	Bicarbonate	continuous
Bilirubin	continuous	Calcium	continuous
Total CO2	continuous	Chloride	continuous
Creatinine	continuous	Glucose	continuous
Hematocrit	continuous	Hemoglobin	continuous
INR(PT)	continuous	Lactate	continuous
MCH	continuous	MCHC	continuous
MCV	continuous	Magnesium	continuous
PT	continuous	PTT	continuous
Phosphate	continuous	Platelet Count	continuous
Potassium	continuous	RDW	continuous
Red Blood Cells	continuous	Sodium	continuous
Urea Nitrogen	continuous	White Blood Cells	continuous
pCO2	continuous	pH	continuous
pO2	continuous	JH-HLM	continuous
Dyspnea Assessment	continuous	Daily Weight	continuous
Glucose	continuous	Heart Rate	continuous
DBP	continuous	SBP	continuous
O2 Flow	continuous	O2 Sat (%)	continuous
Pain Level	continuous	Pain Level Response	continuous
Phosphorous	continuous	Respiratory Rate	continuous
Richmond-RAS Scale	continuous	Temperature (°F)	continuous

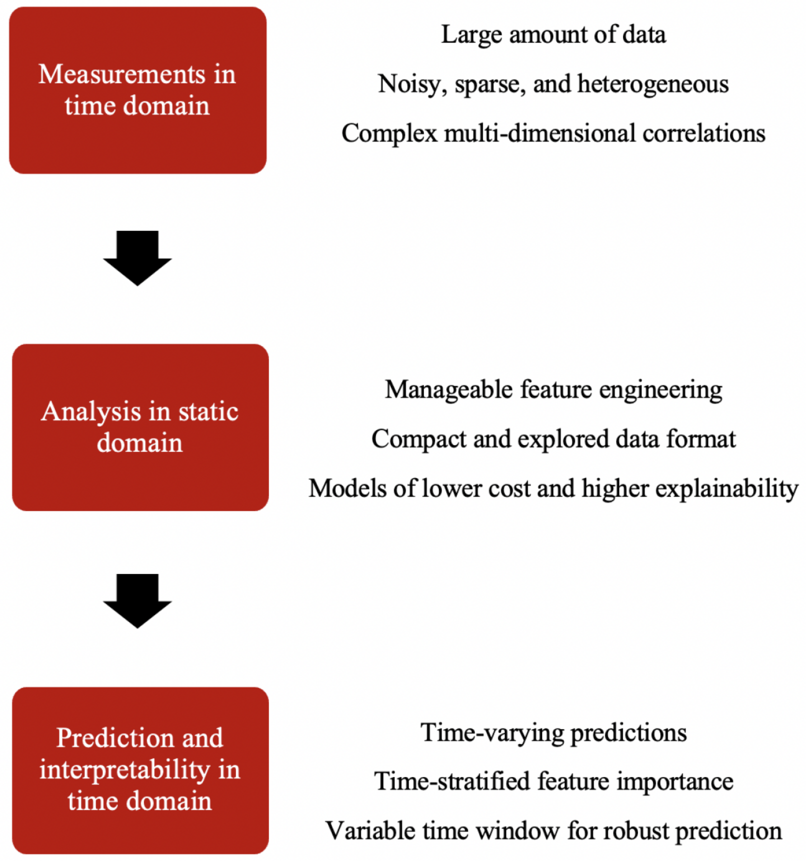

Figure S4: Proposed paradigm shift for pseudo-dynamic prediction with time-series data. Instead of relying only on opaque, costly, and slow deep learning models to offer time-stratified prediction without clinically relevant feature engineering, we break the problem into several non-overlapping time windows of consistent length each of which is treated as a tabular prediction problem to be solved with predictive and interpretable machine learning models. Because predictions are given for different time points, the risk predictions vary over time for each patient thereby providing pseudo-dynamic prediction from time-series data.

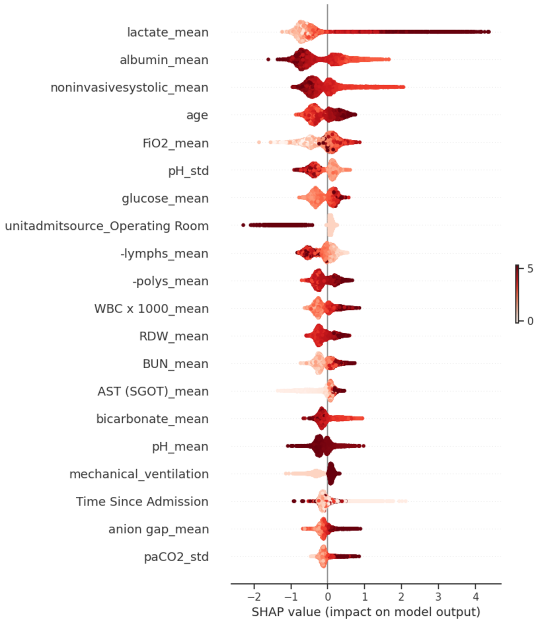

(a) Importance of clinical variables for mortality prediction across patient ICU stay

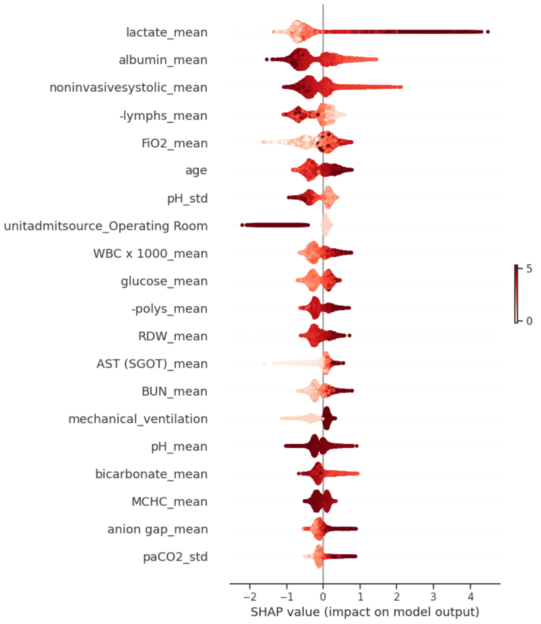

(b) Importance of clinical variables for mortality prediction across patient ICU stay with Gaussian noise added

Figure S5: SHAP values of features for XMI-ICU prediction of mortality and with random noise added. No significant change appears in the top features satisfying the perturbation constraints. The relative vertical ranking of the features corresponds to higher importance of those features in making a correct prediction. The darker colours in the horizontal plane for each feature correspond to higher values of that feature contributing to either stronger positive prediction (if darker colour on the right side of the vertical line) or stronger negative prediction of outcome otherwise.

Table S4: Machine Learning Methods Deployed During Study

**Models**	**Brief Description**
Logistic Regression	Maps a linear relationship taking into account correlations between covariates
Linear Discriminant Analysis	Maps a linear relationship assuming the covariates are independent and normally distributed
Naive Bayes<br>ignoring correlations	A probabilistic estimator assuming conditional independence between covariates
Random Forest	An ensemble of decision trees whose predictions are aggregated for the final prediction
XGBoost	Using extreme gradient-boosting to improve ensembles of random forests for prediction
Ensemble<br>in an ensemble	Using AdaBoosted decision trees, similar to XGBoost but with different boosting mechanism,
Ensemble with XGBoost	Using our XGBoost as the base estimator in the ensemble hierarchy instead of AdaBoost

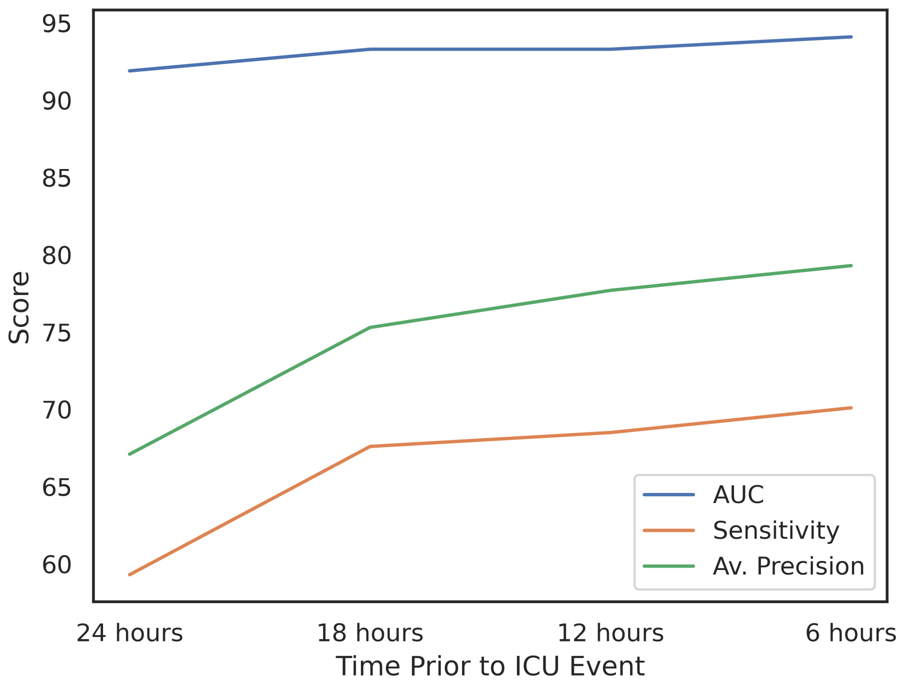

Figure S6: XMI-ICU performance across time for mortality prediction as evaluated on MIMIC-IV held-out test sets after training on an internal MIMIC-IV set

Table S5: AUROC test results for XMI-ICU evaluated on subpopulations for 6 hour prediction.

	Mortality	Mortality (external MIMIC-IV)
Men	90.2	81.9
Women	92.7	77.5
Caucasian	91.7	81.8
Black/Hispanic	92.3	75.6

Table S6: Validation (Val: Mean ± SD) and test prediction results for mortality prediction 6 hours in advance.

	Val AUC	AUC	Average Precision
XMI-ICU	**92.9 ± 0.4**	**91.9**	68.7
APACHE IV	-	69.8	31.9
TabNet	86.8 ± 2.1	85.0	64.7
TabNet (pretrained)	-	83.1	**82.1**
NODE	87.8 ± 0.7	86.3	66.3
Logistic Regression	91.3 ± 0.4	90.1	61.5
Random Forest	92.1 ± 0.5	91.1	64.4
SVM	91.3 ± 0.8	90.2	62.1
SVM (linear)	88.5 ± 0.7	88.6	67.8
LDA	80.5 ± 2.0	78.6	33.3

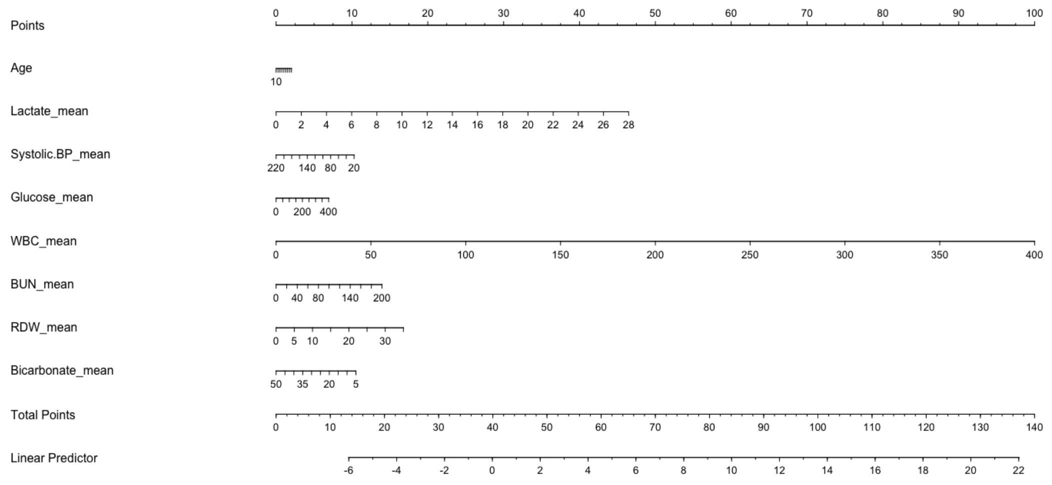

Figure S7: Nomogram to estimate the risk of mortality in MI patients in multi-centre ICUs from the eICU test set. The nomogram includes the top 8 features identified by the model as highly predictive for this patient population as well as the external cohort. The nomogram is used to provide insight into risk calculation based on these features using ranges measured for the patient. One simply draws a straight line from each feature value to the points line, then points are added on the total points line after which a straight line is drawn downward to the linear predictor for a risk estimate respectively. The risk score calculated through this nomogram is for 24-hour prediction.
